# Changing diagnostic criteria for gestational diabetes in Sweden - a stepped wedge national cluster randomised controlled trial - the CDC4G study protocol

**DOI:** 10.1186/s12884-019-2547-5

**Published:** 2019-11-01

**Authors:** Helena Fadl, Maryam Saeedi, Scott Montgomery, Anders Magnuson, Erik Schwarcz, Kerstin Berntorp, Verena Sengpiel, Elisabeth Storck-Lindholm, Helena Strevens, Anna-Karin Wikström, Sophia Brismar-Wendel, Martina Persson, Stefan Jansson, Fredrik Ahlsson, Carina Ursing, Linda Ryen, Kerstin Petersson, Ulla-Britt Wennerholm, Karin Hildén, David Simmons

**Affiliations:** 1 Department of Obstetrics and Gynaecology, Faculty of Medicine and Health, Örebro University, Örebro, Sweden; 2 Clinical Epidemiology and Biostatistics, School of Medical Sciences, Örebro University, Örebro, Sweden; 3 Clinical Epidemiology Unit, Department of Medicine, Karolinska Institutet, Stockholm, Sweden; 4 Department of Epidemiology and Public Health, University College London, London, UK; 5 Clinical Epidemiology and Biostatistics, University Hospital Örebro, Örebro, Sweden; 6 Department of Internal Medicine, School of medical health and sciences, Örebro University Hospital, Örebro, Sweden; 7 Department of Endocrinology, Skåne University Hospital, Clinical Research Center Malmö, Lund University, Lund, Sweden; 8 Department of Obstetrics and Gynaecology, Sahlgrenska University Hospital, Sahlgrenska Academy, University of Gothenburg, Gothenburg, Sweden; 9 Department of Obstetrics and Gynaecology, Södersjukhuset, Stockholm, Sweden; 10 Department of Obstetrics and Gynaecology, Skåne University Hospital, Clinical Research Center Lund, Lund University, Lund, Sweden; 11 Women’s and Children’s Health, Uppsala university, Uppsala, Sweden; 12 Department of Clinical Sciences, Karolinska Institutet Danderyd Hospital, Stockholm, Sweden; 13 Department of Paediatrics, Sachsska Children’s and Youth hospital and Department of Clinical Science and Education, Karolinska Institute, Stockholm, Sweden; 14 School of Medical Sciences, University Health Care Research Center, Örebro University, Örebro, Sweden; 15 Department of Women’s and Children’s health, Uppsala University, Uppsala, Sweden; 16 Department of Endocrinology, Södersjukhuset, Stockholm, Sweden; 17 Center for Health Care Science, Faculty of Medicine and Health, Örebro University, Örebro, Sweden; 18 Department of Clinical Sciences, Obstetrics and Gynaecology, Umeå University, Umeå, Sweden; 19 Department of Obstetrics and Gynaecology, Institute of Clinical Sciences, Sahlgrenska Academy, University of Gothenburg, Sahlgrenska University Hospital, Gothenburg, Sweden; 20 Macarthur Clinical School, Western Sydney University, Campbell town, Australia; 21 Faculty of Medicine and Health, Örebro University, Örebro, Sweden

**Keywords:** Gestational diabetes mellitus, Pregnancy outcomes, Diagnostic criteria, WHO 2013 criteria, Stepped wedge cluster randomised controlled trial, LGA, Health economics, Obesity

## Abstract

**Background:**

The optimal criteria to diagnose gestational diabetes mellitus (GDM) remain contested. The Swedish National Board of Health introduced the 2013 WHO criteria in 2015 as a recommendation for initiation of treatment for hyperglycaemia during pregnancy. With variation in GDM screening and diagnostic practice across the country, it was agreed that the shift to new guidelines should be in a scientific and structured way. The aim of the Changing Diagnostic Criteria for Gestational Diabetes (CDC4G) in Sweden (www.cdc4g.se/en) is to evaluate the clinical and health economic impacts of changing diagnostic criteria for GDM in Sweden and to create a prospective cohort to compare the many long-term outcomes in mother and baby under the old and new diagnostic approaches.

**Methods:**

This is a stepped wedge cluster randomised controlled trial, comparing pregnancy outcomes before and after the switch in GDM criteria across 11 centres in a randomised manner. The trial includes all pregnant women screened for GDM across the participating centres during January–December 2018, approximately two thirds of all pregnancies in Sweden in a year. Women with pre-existing diabetes will be excluded. Data will be collected through the national Swedish Pregnancy register and for follow up studies other health registers will be included.

**Discussion:**

The stepped wedge RCT was chosen to be the best study design for evaluating the shift from old to new diagnostic criteria of GDM in Sweden. The national quality registers provide data on the whole pregnant population and gives a possibility for follow up studies of both mother and child. The health economic analysis from the study will give a solid evidence base for future changes in order to improve immediate pregnancy, as well as long term, outcomes for mother and child.

**Trial registration:**

CDC4G is listed on the ISRCTN registry with study ID ISRCTN41918550 (15/12/2017)

## Background

Hyperglycaemia during pregnancy is a growing problem globally and is associated with several long- and short-term adverse outcomes for the mother and offspring [1–5]. In particular, maternal hyperglycaemia induces foetal hyperinsulinemia with enhanced foetal growth and increased risk of foetuses being born large for gestational age (LGA). The rate of macrosomia, neonatal hypoglycaemia and caesarean delivery increase linearly with increasing levels of maternal hyperglycaemia [6]. Women with GDM are also at higher risk of gestational hypertension and preeclampsia. In the long term, metabolic diseases such as Type 2 diabetes mellitus (T2DM), cardiovascular disease and obesity are more frequent among women with prior GDM [2, 7–9] . In offspring GDM has been shown to be associated with adiposity and risk for prediabetes [3, 4, 10, 11]. Treatment of GDM involves dietary and physical activity advice, blood glucose monitoring, and where necessary metformin or/and insulin therapy. GDM management has been shown to reduce maternal and perinatal morbidity [12–14].

Internationally, the prevalence of GDM varies from 1 to 28% depending on the ethnic composition and prevalence of T2DM of the background population, local GDM screening strategies and diagnostic criteria [15–17]. This variation in screening and diagnostic approaches has made global comparisons of GDM prevalence and outcomes problematic. In order to progress towards a universal standard approach to GDM diagnosis, the World Health Organisation (WHO) recommended in 2013 that a 2-h 75 g oral glucose tolerance test (OGTT) should be used, with three time points blood testing (fasting, 1- and 2-h) [1]. These 2013 WHO criteria define GDM as ≥5.1, ≥10.0 and/or ≥ 8.5 mmol/L fasting, 1-h and/or 2-h thresholds. These cut-off values are based on a ≥ 75% adjusted excess risk of adverse neonatal outcomes (e.g. large for gestational age (LGA), foetal hyperinsulinemia), based on data from the Hyperglycaemia and Adverse Pregnancy Outcomes (HAPO) study involving 25,505 women from nine countries [18]. There has been debate over the merits of these new criteria as the number of women diagnosed with GDM would be expected to increase by 15–30%, raising concerns over their cost and clinical effectiveness [19–22]. While the thresholds are based upon calculated excess risk, the decision to base the diagnostic criteria on ≥75% excess risk was based upon consensus. Some countries (e.g. Canada, Norway) have reduced GDM numbers by using thresholds based upon a ≥ 100% excess risk of adverse neonatal outcomes (5.3- and/or 10.6- and/or 9.0 mmol/L, respectively) [23, 24]. Others have not based their criteria on HAPO data at all (e.g. England, New Zealand).

Older Swedish GDM criteria are based on varying cut-off values. If a fasting threshold was used (not used in one region), then ≥7.0 mmol/L was considered GDM. The 2 h criteria ranged from 9.0–11.1 mmol/L, using either capillary or venous samples. Using these criteria, 1–3% of the 115, 000 births were complicated by GDM annually. In June 2015, following a review of the available evidence, the Swedish National Board of Health and Welfare (SNBHW) recommended a move to the 2013 WHO diagnostic criteria, using venous sampling. The SNBHW made no recommendations in relation to the screening (e.g. universal vs risk factor).

With the current variation in GDM screening/diagnostic practice across Sweden [25], and the debate over the criteria, there was a recognition that the transition to the recommended new guidelines could be either by an ad hoc, or planned and structured way, to minimise clinical variation. National registers in Sweden offer a possibility to assess the impact of introducing the new GDM criteria on pregnancy outcomes and long-term health for both mother and child. A stepped wedge cluster randomised controlled trial (SW-CRCT) was the most realistic approach to evaluate this change on a national level [26–30]. A SW-CRCT involves randomly allocated times for clusters to introduce an intervention, allowing participants before and after any change to serve as control and intervention groups respectively. Reasons for choosing this study design were:

1. The intervention is a change in one clinical routine in a population, making it unrealistic to randomise by individual.
2. All sites were to end with adopting the national guidelines as promptly as possible, which would have been delayed using a simple case control approach.
3. Roll out in one go, across multiple centres was unrealistic, making a parallel cluster randomised controlled trial unrealistic. Steady adoption, site by site, was seen as the best way to make the change in the most organised manner.

This study will provide evidence to help address some of the clinical controversies over GDM diagnosis. Our aim is to test whether there is a reduction in adverse neonatal and maternal outcomes following the implementation of the new GDM criteria, and to evaluate the health economic impact on a population level.

Our specific objectives are:

1. To compare the rates of LGA and other adverse neonatal and maternal pregnancy outcomes before and after the change in GDM diagnostic criteria.
2. To compare the health care costs before and after the change and assess the net costs/savings.
3. To create prospective cohorts to compare the long-term outcomes in mothers and offspring exposed to the old and new diagnostic approaches.

## Methods

### Study design

**Fig. 1:**
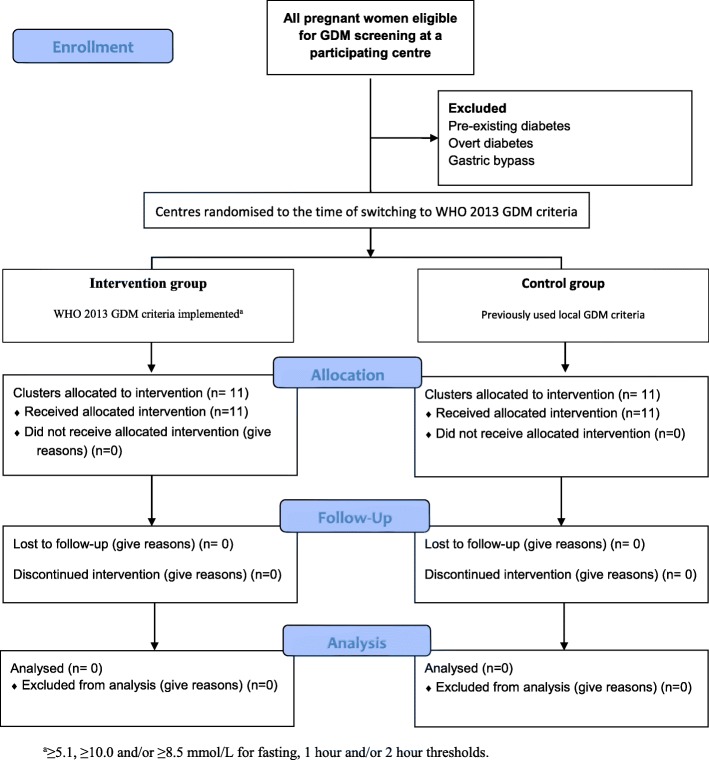
Time of preparation, set up, control and intervention in the CDC4G study during 2017, 2018 and 2019. Q = quartile, 3 months period

**Fig. 2:**
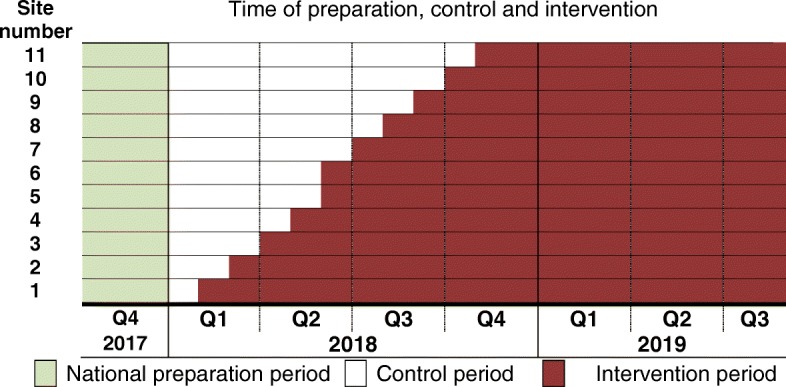
CONSORT 2010 flow diagram of the stepped wedge cluster randomised CDC4G - trial

The Changing Diagnostic Criteria for Gestational diabetes (CDC4G) in Sweden study is a national prospective, unblinded, SW-CRCT of the switch from the former Swedish diagnostic criteria to the WHO 2013 criteria for GDM i.e. to the 3 point OGTT with fasting plasma glucose, 1-h and/or 2-h diagnostic thresholds of ≥5.1, ≥10.0, ≥8.5 mmol/L, respectively (Fig. 1). Each participating centre constitutes one cluster, in which the patients continue to undergo screening for GDM following their usual approach, see Table 1. The time of transition to the new criteria is randomised and subsequently rolled out until all 11 clusters (i.e. centres) implement the new GDM regimes during 2018, see Fig. 2. The transition necessitated the introduction of a fasting glucose sample in one region, and a 1-h sample across all 11 regions. This means that a total population evaluation of the change is required, as many women with GDM diagnosed by the new criteria, will not be identified during the control period (e.g. those without the 1-h test). A sub-analysis among those with untreated and treated GDM by the WHO 2013 is also planned.

**Table 1:** List of included regions and methods for diagnosing GDM

Centres	Number of births 2017^a^	Indication for a diagnostic OGTT									Diagnostic criteria prior to switch (mmol/L)	Method for glucose analysis
		Previous				BMI at booking	FH^d^	Polyhyd ramnios	Accelerated fetal growth	RPG		
		GDM	Macro somia^b^	LGA^c^	Stillbirth							
1. Stockholm	28,602	T1 & 24–28	24–28			≥35		Yes	Yes	RPG^e^ ≥ 9 mmol/L at booking, week 25, 29, 32/33, 37/38.	FPG < 7.0 and 2-h PG 8.9–11.1	Roche Cobas Beckman Coulter Au. Siemens Advia (hexokinase)
2. Örebro	3565	24–28	24–28			≥35	24–28	Yes	Yes-within 3 days	RPG^e^ ≥ 9 mmol/L at booking, week 24, 28/29, 33, 37.	FPG < 7.0 and 2-h PG 8.9–11.0	Siemens Advia (hexokinase)
3. Västmanland	3120	24–28	24–28			> 35		Yes	Yes	RPG^e^ ≥ 9.0 mmol/L at booking week 25, 30, 35.	FPG < 7.0 and 2-h PG 8.9–11.	Beckman Coulter Au (hexokinase)
4. Dalarna	3232	12–14/24–28	24–28	24–28		> 35	24–28	Yes	Yes	RPG^e^ ≥ 9.0 mmol/L at booking, week 24, 28/29, 33, 37.	FPG < 7.0 and/or 2-h PG 8.9–11.1	Siemens Advia (hexokinase)
5. Uppsala	4200	12–14/24–28	24–28	24–28		≥35	24–28	Yes	Yes	RPG^e^ ≥ 8.8 mmol/L at booking, week 25, 28/29, 33, 37	FPG ≥7.0 and/or 2-h PG ≥10.0	Abbott Architect (hexokinase)
6. Göteborg	9550	25–29	25–29	25–29		≥35	25–29	Yes	(Yes)	Within 1 week if RPG^e^ 8.0–12.1 mmol/L at first antenatal care visit, week 25, 28–29, 35–36.	FPG ≥7.0 and/or 2-h PG ≥10.0 RPG^c^ ≥ 12.2	Nova Biomedical StatStrip TM Multi-Well™ (glucose oxidase)
7. Gotland	538	24–28	24–28		24–28	≥35	24–28	Yes	Yes	RPG^e^ ≥ 9.0 mmol/L at enrollment week 25, 29, 32/33, 37/38.	FPG < 7.0 and 2-h PG 8.9–11.0	HemoCue AB HemoCue 201 RT (glucose dehydrogenase)
8. Halland	4446	12/ 24–28		24–28	24–28	≥30	24–28	Yes	Yes	RPG^e^ ≥ 8.0 mmol/L at enrollment, week 12, 28/29, 32, 37	FPG < 7.0 and 2-h PG 9.0–11.1	HemoCue AB HemoCue 201 RT (glucose dehydrogenase)
9. Malmö	4944	10–12	10–12			≥35	10–12	Yes	No	Capillary 75 g OGTT week 28 in all women. FPG^e^ ≥ 7 and/or 2-h PG^e^ ≥ 10.0 mmol/L indication for a diagnostic OGTT	FPG ≥7.0 and/or 2-h PG ≥9.0	Roche Cobas (hexokinase)
10. Lund	3703	10–12	10–12			≥35	10–12	Yes	No			
11. Kristianstad	2085	10–12	10–12			≥35	10–12	Yes	No			

Legend: When not otherwise stated, glucose measurement is based on venous plasma

*BMI* body mass index, *FH* family history, *FPG* fasting plasma glucose, *IUFD* intrauterine fetal death, *LGA* large for gestational age, *OGTT* oral glucose tolerance test, *PG* plasma glucose, *RPG* random plasma glucose, *T1* trimester 1

^a^Number of births per year based on data from the Swedish Medical Birth register year 2017

^b^Defined as birth weight ≥ 4.5 kg

^c^Defined as birth weight ≥ + 2 standard deviations above the Swedish reference curve [31]

^d^In Dalarna, Malmö, Lund, Kristianstad, Uppsala, Gotland, Halland defined as first degree relative with type 1 or type 2 diabetes, otherwise first degree relative with type 2 diabetes

^e^Based on capillary samples

Sites have previously varied in how samples were taken, with some using capillary and some venous sampling. As venous sampling was used in the HAPO study, and is the approach recommended by the SNBHW and the WHO, all study centres using capillary sampling for diagnosing GDM changed to venous during the national preparation period (September–December 2017). GDM clinical management has also varied across the country and new guidelines for GDM treatment and obstetric surveillance were introduced during the national preparation period, prior to the commencement of the trial. It was agreed that no local policy should change over the trial period.

The trial was performed within the Swedish Network for Clinical Studies within Obstetrics and Gynaecology (SNAKS; www.sfog.se).

#### Participants

All regions in Sweden were invited to participate over the 12-month study period. Eleven (65–67,000 birth/year) out of 21 regions in Sweden agreed to participate. All women within the participating regions (including within both primary and secondary care) across Sweden are included in the study, unless they opt out from the national register. Women with pre-existing diabetes are excluded. This approach, to use de-identified data from the national registers, without individual consent, was approved by the Uppsala Regional Ethics committee on behalf of all Research Committees across Sweden. The national SPR uses an opt out approach, with less than 100 opts out yearly.

#### Outcomes

The primary outcome is LGA (birth weight above the 90th percentile for gestational age and sex using centile tables) based on Swedish reference population (twins, major malformations and intrauterine deaths excluded from reference population). This is the only common pregnancy outcome shown to be substantially and consistently reduced by treating GDM [32].

Secondary outcomes for neonate and mother are listed in Table 2.

**Table 2:** Outcomes, sampling and measurements

			Data collection points		
	Method	Sources	Baseline	During pregnancy	Birth/postpartum
Primary Outcome					
LGA^a^	scale	SPR^b^			x
Secondary maternal outcomes					
Gestational Hypertension	Blood pressure or ICD code	SPR^b^/ eCRF^f^		x	x
Pre-eclampsia	ICD code	SPR^b^		x	x
Shoulder dystocia	ICD code	SPR^b^			x
Induction of labour	ICD code	SPR^b^			x
Emergency LSCS	ICD code	SPR^b^			x
Elective LSCS	ICD code	SPR^b^			x
Instrumental delivery	ICD code	SPR^b^			x
Length of maternal stay from delivery to discharge	hours, days	SPR^b^			x
Perineal trauma-3 and 4 degree	ICD code	SPR^b^			x
Breastfeeding at hospital discharge	Yes/no	SPR^b^			x
Maternal HbA1c (mmol/mol)	Blood sample	eCRF^f^	x	x	
Self-reported health	Scale 1–6	SPR^b^		x	x
Satisfaction with childbirth	Scale 1–10	SPR^b^			x
Post-part bleeding	ml	SPR^b^			x
Gestational weight gain	g or kg	SPR^b^	x	x	
Secondary neonatal outcomes					
Stillbirth (≥22 + 0)	ICD code	SPR^b^/SNQ^c^			x
Neonatal death (< 28 days)	ICD code	SPR^b^ /SCB^d^			x
Erbs palsy	ICD code	SPR^b^/SNQ^c^			x
Metabolic acidosis^g^	Umbilical artery and venous blood sample	SPR^b^			x
1,5,10 min Apgar score < 4	Clinical examination	SPR^b^			x
Hypoxic ischemic encephalopathy I-III	ICD code	SPR^b^/SNQ^e^			x
Intracranial haemorrhage	ICD code	SPR^b^/SNQ^e^			x
Meconium aspiration syndrome	ICD code	SPR^b^/SNQ^e^			x
Mechanical ventilation		SPR^b^/SNQ^e^			x
Fractured clavicle/humerus	ICD code	SPR^b^/SNQ^e^			x
Blood glucose in the infants (mmol/L)	Blood sample	eCFR^f^			x
Preterm birth < 37, 34 or 32 weeks	ICD code	SPR^b^			x
NICU date	(xxxx/yy/zz)	eCRF^f^			x
NICU days	days	eCRF^f^/SPR^b^			x
SGA^h^	Calculated from birth weight / gestational age/gender	SPR^b^			x
Bilirubin (highest value)	Blood sample	eCRF^f^			x
Phototherapy	Medical record	eCRF^f^			
Hypoglycaemia needing IV therapy		eCRF^f^			x
Supplementary feeding					
Supplementary feeding indication	Medical record	eCFR^f^			x
Interval (hours) and volume (ml) of supplementary feeding		eCFR^f^			x
Product used		eCFR^f^			x
Use of dextrose gel or intravenous treatment		eCFR^f^			x

^a^ Large for Gestational Age: Birth weight above the 90th percentile for gestational age in the Swedish reference population corrected for gestational age and gender

^b^ Swedish pregnancy register: Diagnosis based on ICD codes or values (ml, kg, g, numbers). Variable list available on https://www.medscinet.com/gr/forskare.aspx

^c^ The Swedish National Patient Register

^d^ Statistics Sweden

^e^ Swedish Neonatal Quality Register

^f^ eCFR.www.medscinet.com

^g^ pH < 7.05 and BE > 12 mmol/L or pH < 7.0

^h^ Birth weight < 10th percentile for gestational age, corrected for gestational age and gender. Swedish reference population

A healthcare cost-utility analysis will be undertaken.

#### Recruitment

Women will be recruited de facto by being under the care of a participating health service. Women always have an option to decline testing and, if GDM is diagnosed, to decline treatment. Informed consent will not be requested beyond the routine invitation to opt out of the SPR and the option at any time to refuse any aspect of management.

#### Randomisation

As the expected number of births varies across the participating centres, a stratified randomisation by centre size was conducted using two strata. The first strata was the two largest populated study centres (Stockholm and Gothenburg) which was randomised to change GDM criteria in June or August of 2018. The second strata with the nine remaining centres was changing, one centre per month, in a randomised order from February to July and September to November of 2018. The randomisation allocation was performed using computer-generated, random allocation sequences using SPSS version 22 (Armonk, NY:IBM Corp) by the study statistician at Clinical Epidemiology and Biostatistics, Region Örebro County. The randomisation was concealed from the participating centre and the list is stored in a safe at Örebro University Hospital.

The study statistician provided information on the randomisation to the study coordinator who in turn informed the relevant centre through the local PI, 2 months prior to when the centre being randomised to using the new GDM criteria. Staff at all sites were blinded until informed by their local PI of their start date to change from the *control period* (old GDM criteria) to the *intervention period (*new GDM criteria*)*.

#### Monitoring of adhesion to the study protocol

Every month each centre reported their monitoring according to a checklist on the website (ref according to guidelines) to make sure that the guidelines for the CDC4G study (e.g. GDM treatment, blood sampling methods, obstetric surveillance, switch to the new criteria) were followed every step of the study. In addition, the number of women with GDM diagnosed every month during the study period is registered. Severe possible adverse outcomes such as (severe maternal hypoglycaemia (low blood glucose levels that requires assistance from another person to treat) and lactic acidosis in metformin treated women) is reported each month. Interim analysis is performed by the study coordinator every month to identify any safety or protocol breaches. The study data and safety monitoring board (DSMB) is available to determine whether any safety issues warranted termination of the trial.

#### Procedures

A 3-month *national preparation period* was organized between October to December 2017 (Fig. 2), for all sites to consolidate their local approach to screening, changing diagnostic testing to venous sampling, modifying and consolidating the agreed approach to GDM treatment and the agreed approach to obstetric management.

The trial started on the 1st of January 2018 with 1 month of baseline data collection when no randomisation occurred. Subsequently, at periodic time points called “steps”, sites changed to the new GDM criteria in a randomised order over a 10 months period. In December 2018 all sites used the new criteria. The last birth is expected in August 2019.

##### Booking screening

Booking screening varied between the centres. In some centres, high risk women underwent an OGTT, other centres used universal random blood glucose screening to identify those for referral to OGTT, see Table 1. The OGTT thresholds used for overt diabetes in pregnancy are listed in Table 1.

##### The oral glucose tolerance test (OGTT)

Throughout the study period, a 2-h 75 g OGTT was undertaken following booking screening, at 24–29 weeks gestation or if there was a clinical indication (e.g. polyhydramnios, accelerated growth or high random blood glucose).

Venous sampling were used with glucose measurements performed either by the local laboratory or with point of care testing (Table 1). All the laboratories are accredited according to SS-EN-ISO/IEC 15189. As part of the accreditation the laboratories participate in inter-laboratory comparison schemes either from Equalis (https://www.equalis.se/en/) or in schemes organised by the local hospital organisation.

##### GDM management guidelines

GDM management (treatment and obstetrical surveillance) was the same in all centres before and after the switch. All women received a GDM information leaflet and written dietary advice in Swedish and translated into five other languages (English, Pashto, Somali, Arabic and Dari). All women diagnosed with GDM were offered lifestyle advice from a registered dietitian. Further general GDM information was provided by the midwife if lifestyle treated, and the diabetes team if treated with insulin or metformin. A local diabetes nurse or trained midwife taught women on how to undertake self-measurement of plasma glucose (SMPG). Different meters were used, but all were capable of uploading data through DIASEND® (Glooko AB, Göteborg, Sweden) which is considered to be part of the local clinical records. SMPG was recommended to occur four times per day: Fasting and 1 h after breakfast, lunch and dinner among those who are lifestyle treated and seven times per day among those treated with insulin and/or metformin (before and 1 h after meals and at night before bed).

Target values for capillary plasma glucose were < 5.3 mmol/L fasting (before breakfast), < 6.0 mmol/L before other meals, < 8.0 mmol/L 1 h after meals, and < 7.0 mmol/L before bedtime. Those with three or more values above target during 1 week were commenced on pharmacological treatment or intensified treatment if already on metformin/insulin. GDM management (treatment and obstetrical surveillance) was the same in all centres before and after the switch.

### Pharmacological treatment

Metformin is accepted for treatment of GDM by the Swedish National Board of Health (https://www.socialstyrelsen.se/globalassets/sharepoint-dokument/artikelkatalog/nationella-riktlinjer/2015-4-12.pdf) and it is used mainly for overweight patients (BMI ≥ 25 kg/m^2^), especially where fasting glucose and basal glucose levels were elevated during the day. Written and oral information was provided regarding adverse effects and that metformin crosses the placenta. Start dose was 500 mg once daily, with increasing one tablet every third day until target glucose level was achieved.

Insulin is recommended when metformin is not expected to bring hyperglycaemia rapidly under control, or when considered inappropriate for clinical reasons or declined by the patient. If fasting blood glucose is above the target, intermediate acting insulin (NPH) is the first line of choice. Long acting analogue insulins are considered if the blood glucose targets are not reached. Rapid acting insulin analogues are used to manage elevated postprandial glucose levels. Evaluation of glucose values and titration of insulin dose are recommended twice a week initially, after which titration is performed once a week. Changes in insulin doses occurred if three or four values are above the target value over a 1-week period. Starting doses are 4–8 units depending on the glycaemia and other clinical factors (e.g. BMI).

### Obstetric surveillance

All women with GDM receive written information about health and exercise and continue with the usual obstetric/midwifery care including additional intervention when indicated. Women with diagnosed GDM and lifestyle management alone received expectant care as per normal pregnancy management with foetal weight estimation at week 38 for pre-delivery assessment. For women receiving metformin or insulin treatment, additional ultrasound assessments for foetal weight estimation was performed at least 2 times during pregnancy: at weeks 28–32 and latest at 38 weeks. Induction occurs if not delivered before 40 + 6 for medically treated GDM women, and for diet treated GDM induction at the latest 42 + 0.

### Data collection

Data will be collected through the SPR, a national quality registry [33]. An additional electronic case record form is created with manually entered data not otherwise collected in the SPR (e.g. neonatal blood glucose and repeat OGTT values). Follow up studies will use the National Diabetes Register (NDR) and other Swedish health care registers (SNQ, Prescribed Drug register, Statistics Sweden). Glucose monitoring and target adherence were collected through the use of DIASEND®.

First data set extraction is planned to be completed during 2020. The project coordinator and steering group will have access to the complete dataset when available.

### Statistics and data analysis

#### Sample size and power calculation

The sample size estimation was performed by the study statistician for a stepped wedge cluster randomised design using STATA release 14 [34]. With 11 centres (clusters) participating and an intra cluster correlation (ICC) of 0.0026 a minimum sample size of 47,916 pregnant women (23,958 before change and 23,958 after change of the new GDM criteria) have 90% statistical power with a 5% significance level to detect an absolute reduction in LGA by 1.5% on a population level (from the existing 10 to 8.5%). ICC was estimated from the variation in LGA incidence in year 2012 between participating centres which varied between 7.7 and 13.3% (0.077–0.133). Assuming that the variation in LGA incidence between centres follows a normal distribution, the variation in LGA incidence were estimated as mean 0.1067 and standard deviation (SD) 0.0164. The coefficient of variation (CV) is 0.154 (SD divided by the mean) and the CV can be expressed as an ICC.

The total estimated number of inclusions (=births) during the inclusion period for the participating centres is approximately 65,000. It is not possible to predict the exact number of births during the study due to the differences in sizes of the centres. Some incompleteness (estimated 10%) of data on maternal characteristics (mainly BMI) is expected. The calculation indicates that there will be sufficient power for the main outcome measure (LGA) even if 1–2 centres would drop out.

The proposed statistical analysis methods for stepped wedge designs will be used including Generalized Estimating Equation (GEE) models or mixed effect logistic models to evaluate the primary outcome, LGA. Mixed effect logistic models will include centres as random effects, which is the cluster and the randomisation unit in this design, and the intervention and time periods as fixed effects, or time as a random effect [35]. Adjustment for time is to compensate for calendar time changes over time in LGA incidences that are not due to the intervention changing from the old to new GDM criteria, but because of potential temporal trends in LGA incidence.

As the randomisation is on the cluster level and not on the participant level, we will adjust for important prognostic predictors for LGA, such as chronic hypertension, mother’s age, BMI, country of mother’s birth and the mother’s smoking behaviour during pregnancy. Stratification with interaction tests by prognostic factors will be used to further investigate if the associations with LGA between the two diagnostic criteria are heterogeneous and the new diagnostic criteria have an even larger impact in some subgroups according to risk factors.

Because of the stepped wedge study design, some recommended design-specific analyses will be conducted such as stratification by time period to evaluate if the intervention association with outcomes are homogenous during the study time period, or heterogeneity within centres between the two interventions. The secondary adverse neonatal and maternal outcomes will be analysed using the same type of methods and considerations as for the primary outcome. A sub-analysis will be conducted comparing those with fasting glucose 5.1–6.9 mmol/L and/or 2-h glucose 8.5–8.9 mmol/L before and after the switch in criteria.

#### Health economic evaluation

In a first health economic analysis, increased intervention costs for new diagnostic criteria will be set against reduced healthcare costs during pregnancy and in connection with childbirth. This cost analysis consists of 1) estimation of standard cost of treatment of GDM, 2) mother’s healthcare utilization during pregnancy and the month after delivery and, 3) the infant’s health care utilization the month after birth. Data are retrieved from the SPR. Cost of health utilization is estimated based on the Swedish national cost per Patient database.

In a second analysis, the mother’s quality of life during pregnancy and after birth will also be included. It will be expressed in quality adjusted life years (QALY) based on a single item of self-rated health transformed to preference-based values using a Swedish experience based preference valuation [36]. Self-rated health data during pregnancy and after birth is retrieved from the SPR where it is already collected.

In a third analysis, long-term consequences will be modelled. Differences in incidence of LGA will be modelled on future health for the child and cost implications based on best available evidence. In the same way, the mother’s future health impact from intervention can be modelled based on medical risk markers. Cost effectiveness will be expressed in cost per QALY.

## Discussion

To the best of our knowledge, the CDC4G study is the first national SW-CRCT to evaluate the implementation of the 2013 WHO criteria for GDM. There are a number of non-randomised retrospective studies comparing outcomes before and after the change in criteria. Small studies in Spain and Taipei have shown improved pregnancy outcomes switching from the American Diabetes Association (ADA) criteria to the IADPSG criteria on a population basis [37–39]. A non-randomised retrospective study in the USA found no benefit in outcomes [40] but substantial changes in clinical and obstetric care occurred at the same time [41]. The few randomised trials of treating GDM [12–14] have all studied different settings and diagnostic criteria, and a randomised study of the effect on outcomes by treatment on a population level does not exist. Sweden has a public health care system with almost 100% clinical attendance and public health registries with high coverage. With the availability of Swedish registers and possibility to link registers by the personal identification number, this setting provides a unique possibility to evaluate the impacts of the 2013 WHO criteria on pregnancy outcomes and long-term health for both mother and child.

The IADPSG recommend universal screening [18], however, in Sweden there has been a diverse approach to screening as shown in Table 1. Screening criteria at each centre did not change through the trial and should not impact on the validity of the SW-RCT. Since universal screening with both fasting and 2-h values has not been the routine; the existing screening approaches does reduce the number of women who will have received treatment for GDM, in spite of some hyperglycaemia. Furthermore, in practice, many midwives find it difficult to adhere fully to the plethora of screening criteria. Therefore, any benefit (or harms) from GDM treatment will be less than would have occurred with universal screening.

### SW-CRCT: strengths and limitations

The stepped wedge randomised controlled design is particularly beneficial when the intervention is likely to do more good than harm and is mainly used to evaluate routine implementation, as is the case for the CDC4G trial. The design is practical where it is not possible to deliver the intervention to all the participants at the same time for logistical, practical or financial reasons. The stepped wedge design is also appropriate for cost-effectiveness analysis of an intervention on a population basis (i.e. when changes across a whole population/service occur), also making it suitable for the CD4G study.

Disadvantages of the stepped wedge design are difficulties in blinding the participants and those delivering the intervention since the step from control to intervention is evident for both. It is also important to note that the stepped wedge design is likely to lead to a longer trial duration than a traditional parallel design, particularly where effectiveness is measured immediately after implementation. Since the implementation of the new criteria is expected to do more good than harm and is going to continue after the study period there are no real disadvantages from the participant’s point of view. Step-wedge trials can be impacted upon by wider secular trends that can differ between the beginning and end of the study period. In CDC4G, the design has included an alignment, and run in, of all significant clinical policies before the trial commenced, and a clear directive not to introduce any service changes during the study period. A major limitation in cluster randomised trials is the need for larger numbers to overcome the ICC.

### Ethics

Random allocation is performed at the cluster, not the individual level, and prior informed consent to randomisation is not feasible and will not be requested. This is justifiable on the basis that this is a SNBHW policy directive and hence management of GDM is changing as a result of the new national recommendation. It would be unethical not to evaluate the impact of the move, and the best approach for this evaluation is by conducting an RCT. Women always have the right under Swedish Law to change clinic and refuse any aspect of care.

As there is an a priori acceptance that the intervention will do more good than harm [42], rather than belief of equipoise, it may be unethical to withhold the intervention from a proportion of the participants, or to withdraw the intervention as would occur in a cross-over design.

### Trial status

The study preparation period started during 2017 and data collection 1st of January 2018. The first centre was randomised to the 2013 WHO criteria 1st of February 2018 and the last 1st of November 2018. The study is ongoing during 2018 and 2019, with first data collection during 2020.

## Data Availability

Not applicable.

## Abbreviations

ADA: American Diabetes Association
BMI: Body Mass Index
CDC4G: Changing the Diagnostic Criteria for Gestational diabetes
eCRF: electronic case report form
GDM: Gestational diabetes mellitus
HAPO: Hyperglycaemia and Adverse Outcomes
IADPSG: The International Association of the Diabetes and Pregnancy Study Groups
ICC: Inter Cluster Correlation
ISRCTN: International Standard Randomised Controlled Trial Number
LGA: Large for gestational age
NDR: National Diabetes Register
OGTT: Oral glucose tolerance test
QALY: Quality-adjusted life-year
QOL: Quality of life
RCT: Randomised Controlled Trial
SMPGN: Self Measurement of Plasma Glucose
SNBHW: Swedish National Board of Health and Welfares
SNQ: Swedish Neonatal Quality Register
SPR: Swedish Pregnancy Register
SW-CRCT: Stepped wedge cluster randomised controlled trial
T2DM: Type 2 diabetes mellitus
WHO: World Health Organisation

## References

[1] WHO. Diagnostic criteria and Classification of Hyperglycaemia First Detected in pregnancy 2013. Available from: http://www.who.int/diabetes/publications/Hyperglycaemia_In_Pregnancy/en/index.html. Access 6 May 2019. 24199271

[2] Kramer CK, Campbell S, Retnakaran R (2019). Gestational diabetes and the risk of cardiovascular disease in women: a systematic review and meta-analysis. Diabetologia..

[3] Lowe WL, Scholtens DM, Kuang A, Linder B, Lawrence JM, Lebenthal Y (2019). Hyperglycemia and adverse pregnancy outcome follow-up study (HAPO FUS): maternal gestational diabetes mellitus and childhood glucose metabolism. Diabetes Care.

[4] Scholtens DM, Kuang A, Lowe LP, Hamilton J, Lawrence JM, Lebenthal Y (2019). Hyperglycemia and adverse pregnancy outcome follow-up study (HAPO FUS): maternal Glycemia and childhood glucose metabolism. Diabetes Care.

[5] Fadl HE, Simmons D (2016). Trends in diabetes in pregnancy in Sweden 1998-2012. BMJ Open Diabetes Res Care.

[6] Metzger BE, Lowe LP, Dyer AR, Trimble ER, Chaovarindr U, Coustan DR (2008). Hyperglycemia and adverse pregnancy outcomes. N Engl J Med.

[7] Lauenborg J, Mathiesen E, Hansen T, Glumer C, Jorgensen T, Borch-Johnsen K (2005). The prevalence of the metabolic syndrome in a danish population of women with previous gestational diabetes mellitus is three-fold higher than in the general population. J Clin Endocrinol Metab.

[8] Bellamy L, Casas JP, Hingorani AD, Williams D (2009). Type 2 diabetes mellitus after gestational diabetes: a systematic review and meta-analysis. Lancet..

[9] Anderberg E, Landin-Olsson M, Kalen J, Frid A, Ursing D, Berntorp K (2011). Prevalence of impaired glucose tolerance and diabetes after gestational diabetes mellitus comparing different cut-off criteria for abnormal glucose tolerance during pregnancy. Acta Obstet Gynecol Scand.

[10] Brown FM, Isganaitis E, James-Todd T (2019). Much to HAPO FUS about: increasing maternal Glycemia in pregnancy is associated with worsening childhood glucose metabolism. Diabetes Care.

[11] Longmore DK, Barr ELM, Lee IL, Barzi F, Kirkwood M, Whitbread C (2019). Maternal body mass index, excess gestational weight gain, and diabetes are positively associated with neonatal adiposity in the pregnancy and neonatal diabetes outcomes in remote Australia (PANDORA) study. Pediatr Obes.

[12] Fadl HE, Gardefors S, Hjertberg R, Nord E, Persson B, Schwarcz E (2015). Randomized controlled study in pregnancy on treatment of marked hyperglycemia that is short of overt diabetes. Acta Obstet Gynecol Scand.

[13] Landon MB, Spong CY, Thom E, Carpenter MW, Ramin SM, Casey B (2009). A multicenter, randomized trial of treatment for mild gestational diabetes. N Engl J Med.

[14] Crowther CA, Hiller JE, Moss JR, McPhee AJ, Jeffries WS, Robinson JS (2005). Effect of treatment of gestational diabetes mellitus on pregnancy outcomes. N Engl J Med.

[15] Feig DS, Hwee J, Shah BR, Booth GL, Bierman AS, Lipscombe LL (2014). Trends in incidence of diabetes in pregnancy and serious perinatal outcomes: a large, population-based study in Ontario, Canada, 1996-2010. Diabetes Care.

[16] Helseth R, Salvesen O, Stafne SN, Morkved S, Salvesen KA, Carlsen SM (2014). Gestational diabetes mellitus among Nordic Caucasian women: prevalence and risk factors according to WHO and simplified IADPSG criteria. Scand J Clin Lab Invest.

[17] Ignell C, Claesson R, Anderberg E, Berntorp K (2014). Trends in the prevalence of gestational diabetes mellitus in southern Sweden, 2003-2012. Acta Obstet Gynecol Scand.

[18] Metzger BE, Gabbe SG, Persson B, Buchanan TA, Catalano PA, Damm P (2010). International association of diabetes and pregnancy study groups recommendations on the diagnosis and classification of hyperglycemia in pregnancy. Diabetes Care.

[19] Weile LK, Kahn JG, Marseille E, Jensen DM, Damm P, Lohse N (2015). Global cost-effectiveness of GDM screening and management: current knowledge and future needs. Best Pract Res Clin Obstet Gynaecol.

[20] Chen PY, Finkelstein EA, Ng MJ, Yap F, Yeo GS, Rajadurai VS (2016). Incremental cost-effectiveness analysis of gestational diabetes mellitus screening strategies in Singapore. Asia Pac J Public Health.

[21] Mission JF, Ohno MS, Cheng YW, Caughey AB (2012). Gestational diabetes screening with the new IADPSG guidelines: a cost-effectiveness analysis. Am J Obstet Gynecol.

[22] Gillespie P, O'Neill C, Avalos G, Dunne FP, Collaborators AD (2012). New estimates of the costs of universal screening for gestational diabetes mellitus in Ireland. Ir Med J.

[23] Booth G, Cheng AY, Canadian Diabetes Association Clinical Practice Guidelines Expert C (2013). Canadian Diabetes Association 2013 clinical practice guidelines for the prevention and management of diabetes in Canada. Methods. Can J Diabetes.

[24] Cheng AY, Canadian Diabetes Association Clinical Practice Guidelines Expert C (2013). Canadian Diabetes Association 2013 clinical practice guidelines for the prevention and management of diabetes in Canada. Introduction. Can J Diabetes.

[25] Lindqvist M, Persson M, Lindkvist M, Mogren I (2014). No consensus on gestational diabetes mellitus screening regimes in Sweden: pregnancy outcomes in relation to different screening regimes 2011 to 2012, a cross-sectional study. BMC Pregnancy Childbirth.

[26] Brown CA, Lilford RJ (2006). The stepped wedge trial design: a systematic review. BMC Med Res Methodol.

[27] de Hoop E, van der Tweel I, van der Graaf R, Moons KG, van Delden JJ, Reitsma JB (2015). The need to balance merits and limitations from different disciplines when considering the stepped wedge cluster randomized trial design. BMC Med Res Methodol.

[28] Ferrara A, Hedderson MM, Albright CL, Brown SD, Ehrlich SF, Caan BJ (2014). A pragmatic cluster randomized clinical trial of diabetes prevention strategies for women with gestational diabetes: design and rationale of the gestational Diabetes’ effects on moms (GEM) study. BMC Pregnancy Childbirth.

[29] Hemming K, Haines TP, Chilton PJ, Girling AJ, Lilford RJ (2015). The stepped wedge cluster randomised trial: rationale, design, analysis, and reporting. BMJ..

[30] Hemming K, Lilford R, Girling AJ (2015). Stepped-wedge cluster randomised controlled trials: a generic framework including parallel and multiple-level designs. Stat Med.

[31] Maršál K, Persson PH, Larsen T, Lilja H, Selbing A, Sultan B (1996). Intrauterine growth curves based on ultrasonically estimated foetal weights. Acta Paediatr.

[32] Poolsup N, Suksomboon N, Amin M (2014). Effect of treatment of gestational diabetes mellitus: a systematic review and meta-analysis. PLoS One.

[33] Stephansson O, Petersson K, Bjork C, Conner P, Wikstrom AK. The Swedish pregnancy register - for quality of care improvement and research. Acta Obstet Gynecol Scand. 2017. 10.1111/aogs.13266 PMC5873375 29172245

[34] Hemming K, Taljaard M (2016). Sample size calculations for stepped wedge and cluster randomised trials: a unified approach. J Clin Epidemiol.

[35] Hemming K, Taljaard M, Forbes A (2018). Modeling clustering and treatment effect heterogeneity in parallel and stepped-wedge cluster randomized trials. Stat Med.

[36] Edlund M, Burstrom L, Gerhardsson L, Lundstrom R, Nilsson T, Sanden H (2014). A prospective cohort study investigating an exposure-response relationship among vibration-exposed male workers with numbness of the hands. Scand J Work Environ Health.

[37] Hung TH, Hsieh TT (2015). The effects of implementing the International Association of Diabetes and Pregnancy Study Groups criteria for diagnosing gestational diabetes on maternal and neonatal outcomes. PLoS One.

[38] Wu ET, Nien FJ, Kuo CH, Chen SC, Chen KY, Chuang LM (2016). Diagnosis of more gestational diabetes lead to better pregnancy outcomes: comparing the International Association of the Diabetes and Pregnancy Study Group criteria, and the Carpenter and Coustan criteria. J Diabetes Investig.

[39] Duran A, Saenz S, Torrejon MJ, Bordiu E, Del Valle L, Galindo M (2014). Introduction of IADPSG criteria for the screening and diagnosis of gestational diabetes mellitus results in improved pregnancy outcomes at a lower cost in a large cohort of pregnant women: the St. Carlos Gestational Diabetes Study. Diabetes Care.

[40] Feldman RK, Tieu RS, Yasumura L (2016). Gestational diabetes screening: the International Association of the Diabetes and Pregnancy Study Groups Compared with Carpenter-Coustan Screening. Obstet Gynecol.

[41] McIntyre HD, Jensen DM, Jensen RC, Kyhl HB, Jensen TK, Glintborg D (2018). Gestational diabetes mellitus: does one size fit all? A challenge to uniform worldwide diagnostic thresholds. Diabetes Care.

[42] Smith PG, Morrow RH. Field trials of health intervention in developing countries: a toolbox/edited by Peter G. Smith, Richard H. Morrow. Field trials of health intervention in developing countries: a toolbox/edited by Peter G Smith, Richard H Morrow. 1996.

